# Healthcare Professional’s Knowledge of the Systemic ABCDE Approach: A Cross-Sectional Study

**DOI:** 10.7759/cureus.51464

**Published:** 2024-01-01

**Authors:** Tahani A Althobity, Amjad M Jawhari, Mohammed G Almalki, Abdulmajeed A Altowairqi, Maryam Dighriri, Ibrahim J Alghamdi, Yasser Al Nofaiey

**Affiliations:** 1 General Practice, Taif University, Taif, SAU; 2 Medicine, Taif University, Taif, SAU; 3 Emergency Medicine, Taif University, Taif, SAU

**Keywords:** saudi arabia., emergency, clinical knowledge, intensive & critical care, abcde approach

## Abstract

Background and aim

Assessing the knowledge of healthcare professionals regarding the Airway, Breathing, Circulation, Disability, and Exposure (ABCDE) approach is crucial since it prioritizes the initial assessment and treatment of patients who are critically ill, regardless of the underlying cause or their age. Since adherence requires knowledge, this study aimed to evaluate the knowledge level of the ABCDE approach among healthcare professionals.

Methods

This cross-sectional study among healthcare professionals was performed from April to August 2023 in Taif City, Saudi Arabia. The study included healthcare professionals employed in departments exposed to patients with critical illnesses and excluded those from other specialties and individuals from outside Taif City. Data was collected via Google Forms using a previously validated questionnaire designed to assess the ABCDE approach knowledge among healthcare professionals. Statistical analysis was conducted using IBM SPSS, version 26.

Results

The study included 242 healthcare professionals with a mean (SD) age of 35.77 (9.93) years. About half of the participants were female (52.5%) nurses (50.8%) and had been working in intensive care units (ICU) and neonate intensive care units (NICU) (41.4%). The mean (SD) of the participants' working experience was 9.28 (8.295) years. The overall mean test score was 52.94 % (SD 16.27). The mean knowledge score among males was significantly higher than females (56.37% vs. 49.85%, respectively) (p-value= 0.001). The mean knowledge score was significantly higher among medical specialists and residents (63.308% and 55.67%) than among nurses (46.34%) (p-value <0.001). Attending an advanced trauma life support course and theoretical lecture significantly impacted the total knowledge score among the participants (p-values= 0.001 and <0.001, respectively). The total knowledge significantly increased with age (r: 0.265, p-value <0.001). Years of experience correlated with total knowledge score; with increasing years of experience, the total knowledge was significantly increased (r: 0.248, p-value <0.001).

Conclusion

The ABCDE approach is a valuable tool for the initial examination and treatment of patients in acute medical and surgical emergencies. The findings indicate that there is a need for further awareness programs and training on the ABCDE approach, as the total knowledge score among healthcare professionals was found to be suboptimal. Further research is needed to assess the association between knowledge level and clinical performance in different healthcare settings within Saudi Arabia.

## Introduction

The ABCDE algorithm is a systematic approach for Airway, Breathing, Circulation, Disability, and Exposure used for assessing and treating patients with critical illnesses. This approach suits all clinical emergencies and can be utilized without requiring equipment. It can also be used in an advanced form upon the arrival of emergency medical services in intensive care units, emergency rooms, or general hospital wards [1]. The recommended first step in post-resuscitation care is the ABCDE approach when spontaneous circulation is returned [2]. When assessing a patient, it's important first to evaluate their airway. If the patient can speak, it indicates that their airway is clear. Next, observe the patient's breathing for any signs of respiratory distress, such as a rapid breathing rate, sweating, paleness, bluish tint to the skin, use of accessory muscles to breathe, abdominal breathing, and needing to sit upright to breathe. Check the patient's circulation by examining the color of their skin for signs of cardiovascular issues, such as paleness or cyanosis. To evaluate an individual's disability, look for other signs of other indications of low cardiac output, such as decreased consciousness. Assess the patient's central nervous system function to evaluate their level of consciousness. Lastly, performing a thorough examination may require the patient to undress while ensuring dignity and preventing hypothermia [3-6].

The ABCDE approach simplifies complex situations, providing an algorithm for assessment and treatment, situational awareness, and minimizing diagnosis and treatment time to save lives [7]. Healthcare professionals have been informed of the ABCDE approach. Previous studies revealed that adherence seems to be suboptimal despite many attempts to increase knowledge concerning the ABCDE algorithms. Olgers et al. reported that only 33% of unstable patients were prioritized with the ABCDE approach by healthcare professionals in the Emergency Department (ED). A study performed by Linders et al. revealed that neonatal healthcare professionals only adhered to the ABCDE approach 31.5%. Similarly, a study by Schoeber revealed that healthcare professionals treating critically ill patients lacked a consistent and sufficient understanding of the ABCDE strategy, leading to variations in their approach [8-10].

In addition, prior studies mostly did not evaluate knowledge, but all in the context of life support courses and not regarding the ABCDE approach. Also, no research has been conducted on the knowledge regarding the ABCDE approach of healthcare professionals in Taif, Saudi Arabia, even though it is crucial for improving patient care. Therefore, this study aimed to evaluate healthcare professionals' theoretical understanding of the different components of the ABCDE approach.

## Materials and methods

Study design

The cross-sectional study was conducted using an interview questionnaire from June to August 2023 in Taif City, Saudi Arabia.

Study population

The study was conducted among healthcare professionals, including residents, medical specialists, and nurses employed in departments exposed to critically ill patients, such as anesthesiology, pediatrics, ED, and neonatal and adult intensive care units. Healthcare professionals from other specialties and individuals outside Taif City were excluded from the study.

Data collection

The study questionnaire was designed based on a previously validated multiple-choice assessment tool of the ABCDE approach [10] on the knowledge of a critically ill patient using the systematic ABCDE approach of healthcare professionals. This assessment does not evaluate cardiopulmonary resuscitation. The questionnaire was translated into English from Dutch. 

The data collection form was designed to illustrate socio-demographic characteristics (age, gender, profession, department, working experience, previously attended life support course, and time interval since the last education) and the knowledge level of the systematic ABCDE approach. The ABCDE approach questions, including 29 questions, examined participants' theoretical knowledge level of several aspects of managing a patient's condition in a medical emergency. These aspects included late signs of circulatory failure, internal bleeding, blood value of the primary survey, Alert, Voice, Pain, and Unresponsive (AVPU) score, the appropriate time for using the ABCDE in the assessment of the patient, several aspects of the ABCDE approach, and diagnosing the tension pneumothorax. Moreover, the problem was treated prior to the actual ABCDE approach, and the patient's circulatory condition and shock signs were examined. The questionnaire was then distributed via interviews.

Statistical analysis

Regarding the total knowledge score calculation, the correct answer received a score of '1', and the incorrect answer received a score of '0'. The total knowledge score was the sum of all questions, with a possible range of 0 to 29. The statistical analysis was done using IBM SPSS, version 26 (IBM Corporation, New York, USA). Categorical variables were described as numbers and percentages. Regarding numerical variables, mean and standard deviation (SD) or median and interquartile range (IQR) are calculated depending on the probability distribution. Mann-Whitney and Kruskal-Wallis tests were conducted to find the association between the independent factors and the total knowledge score among the participants. Spearman's rank correlation coefficient test examined the correlation between the total scores and the numerical values. Analyses-of-variance (ANOVA) with backward elimination regression analyses were performed to evaluate the secondary outcomes. Statistical significance was determined by considering p-values below 0.05.

Ethical considerations

All participants provided verbal consent. Ethical approval was obtained from the Scientific Research Ethics Committee at Taif University, with approval number: 44-311, April 27, 2023.

## Results

The study included 242 healthcare professionals with a mean (SD) age of 35.77 (9.93) years. About half of the participants were females (52.5%) and nurses (50.8%). Most of them (41.4%) have been working in intensive care units (ICU) and neonate intensive care units (NICU). Healthcare providers who have been working in the anesthesia department participated less than other departments in the current study (16.9%). According to courses attended by healthcare professionals, most of them attended advanced cardiac life support followed by advanced pediatric life support (43% and 38.8%, respectively). The mean (SD) of the participants' working experience was 9.28 (8.295) years. Medical specialists showed the highest years of experience among all the participants, with a mean (SD) of 16.98 (9.58) years. The interval since the last education had a mean (SD) of 5.5679 (7.69). The medical specialists followed by nurses had the highest mean (SD) of interval since the last education (8.26 (9.537) and 5.01 (5.748), respectively). All details are illustrated in Table 1.

**Table 1:** Basic characteristics of the study participants EM: Emergency medicine; ICU: Intensive care unit; IQR: Interquartile range; NICU: Neonates intensive care unit; SD: Standard deviation

Age (years)	Mean (SD)	35.77 (9.93)	
	Median (IQR)	34 (14)	
	Min-Max	18 - 75	
Parameters	Category	Total Count (n= 242)	Percentage
Gender	Male	115	47.5
	Female	127	52.5
Department	Anesthesia	41	16.9
	Pediatric	51	21.1
	EM	50	20.7
	ICU	50	20.7
	NICU	50	20.7
Profession category	Medical specialist	64	26.4
	Resident	55	22.7
	Nurse	123	50.8
Previously attended life support courses	No life support education	14	5.8
	Advanced Paediatric Life Support	94	38.8
	European Paediatric Advanced Life support	2	0.8
	Advanced Cardiac Life Support	104	43.0
	Advanced Trauma Life Support	30	12.4
	Pre-Hospital Paediatric Life Support	2	0.8
	Training by department	37	15.3
	Theoretical lecture	17	7.0
	Other life support education	82	33.9
Working experience in years	Total	Mean (SD)	9.28 (8.295)
		Median (IQR)	8 (11)
	Medical specialist	Mean (SD)	16.98 (9.58)
		Median (IQR)	15.5 (13.75)
	Resident	Mean (SD)	3.76 (4.446)
		Median (IQR)	2 (3)
	Nurse	Mean (SD)	7.755 (5.70)
		Median (IQR)	8 (13)
Interval since the last education	Total	Mean (SD)	5.5679 (7.69)
		Median (IQR)	2 (8)
	Medical specialist	Mean (SD)	8.26 (9.537)
		Median (IQR)	5 (9)
	Resident	Mean (SD)	3.459 (8.019)
		Median (IQR)	1 (2)
	Nurse	Mean (SD)	5.01 (5.748)
		Median (IQR)	2 (8)

The total knowledge score of healthcare professionals regarding the ABCDE approach ranged from 3 to 26 out of 29, with a mean (SD) of 15.36 (4.718) and a 95% confidence interval of 14.76: 15.95. As shown in Table 2, the overall mean test score was 52.94 % (SD 16.27). The total score was significantly associated with gender, profession category, and attending some courses. 

**Table 2:** Total ABCDE knowledge score per gender, department, profession category, and attended life support courses EM: Emergency medicine; ICU: Intensive care unit; NICU: Neonates intensive care unit; SD: Standard deviation * Mann-Whitney test; ** Kruskal-Wallis Test

Parameters	Mean (SD) Score	Mean (SD) Score %	95%- Confidence Interval for Mean %	P-value
Overall score (n = 242)	15.36 (4.72)	52.94 (16.27)	50.889 - 55.009	-
Gender				
Male	16.35 (5.16)	56.37 (17.8)	53.08 - 59.66	0.001*
Female	14.46 (4.09)	49.85 (14.1)	47.37 - 52.327	
Department				
Anesthesia	16.56 (4.48)	57.1 (15.44)	52.23 - 61.98	0.051**
Pediatric	15.45 (4.07)	53.279 (14.02)	49.335 - 57.22	
EM	15.72 (5.25)	54.2 (18.11)	49.059 - 59.35	
ICU	13.86 (4.35)	47.79 (14.99)	43.53 - 52.05	
NICU	15.40 (5.11)	53.10 (17.6)	48.09 - 58.11	
Profession category				
Medical specialist	18.36 (3.94)	63.308 (13.59)	59.91 - 66.7	< 0.001**
Resident	16.15 (4.86)	55.67 (16.74)	51.148 - 60.1998	
Nurse	13.44 (4.09)	46.34 (14.136)	43.81 - 48.86	
Previously attended life support courses				
No life support education	12.93 (6.23)	44.58 (21.49)	32.17 - 56.989	0.122*
Advanced Paediatric Life Support	15.87 (4.49)	51.817 (16.7)	49.1- 54.53	0.194*
European Paediatric Advanced Life support	14 (11.31)	48.27586 (39)	-302.24 - 398.79	0.879*
Advanced Cardiac Life Support	15.68 (4.64)	50.97 (15.98)	50.97 - 57.186	0.372*
Advanced Trauma Life Support	18.17 (5.09)	62.64 (17.538)	56.09 - 69.1925	0.001*
Pre-Hospital Paediatric Life Support	19 (0)	65.517 (0)	65.5 - 65.517	0.176*
Training by department	15.89 (4.97)	54.7996 (17.14)	49.08 - 60.51	0.413*
Theoretical lecture	19.53 (3.67)	67.34 (12.67)	60.825 - 73.86	< 0.001*
Other life support education	15.59 (4.64)	53.74 (16)	50.227 - 57.258	0.608*

Regarding gender, the mean knowledge score was significantly higher among males compared to females (56.37% vs. 49.85%, respectively) (p-value= 0.001). Furthermore, the mean knowledge score was significantly higher among medical specialists and residents (63.308% and 55.67%) compared to nurses (46.34%) (p-value <0.001). Attending an advanced trauma life support course and theoretical lecture significantly impacted the total knowledge score among the participants (p-values= 0.001 and <0.001, respectively). However, the department where they worked and attended other courses did not significantly impact the total score.

There was a weak correlation between age and total knowledge score; with increasing age, the total knowledge was significantly increased (r: 0.265, p-value <0.001). Additionally, years of experience showed a weak positive correlation with total knowledge score (r: 0.248, p-value <0.001) as shown in Table 3.

**Table 3:** Correlation between age, years of experience, and last education interval and total ABCDE knowledge score among participants *Spearman's rank correlation coefficient test

Variables	Correlation Coefficient (r)*	P-value
Age	0.265	<0.001
Working experience in years	0.248	<0.001
Interval since the last education	0.095	0.159

After obtaining backward regression analyses, a model was yielded in which working experience and attending advanced trauma life support courses and theoretical lectures significantly affected the total test score (Table 4). Each 1-year increase in working experience yielded an increase in the total score of 0. 135 (CI: 0.050, 0.219, p-value= 0.002). Participants who attended the advanced trauma life support course scored higher than those who did not attend the course by 2.368 (CI: 0.529, 4.207, p-value= 0.012). In addition, attending the theoretical lecture significantly increased the total score by 3.075 (CI: 0.704, 5.445, p-value= 0.011).

**Table 4:** Multiple regression analysis for the factors affecting ABCDE knowledge score N= 242; R2= 0.154; p-value < 0.001

Variables	Regression coefficient (B)	95% Confidence interval	P-value
Gender (0=female, 1= male)	0.673	-0.549, 1.895	0.279
Working experience (years)	0.135	0.050, 0.219	0.002
Interval since the last education (years)	-0.054	-0.142, 0.035	0.233
Advanced Cardiac Life Support (0= no, 1 = yes)	0.686	-0.514, 1.887	0.261
Advanced Trauma Life Support (0= no, 1 = yes)	2.368	0.529, 4.207	0.012
Theoretical lecture (0= no, 1 = yes)	3.075	0.704, 5.445	0.011

## Discussion

The ABCDE approach is a commonly used method for diagnosing and treating critically ill patients of all ages, using a systematic prioritization of fatal disorders. Moreover, the international guidelines and courses recommend using the ABCDE approach [10]. Algorithm adherence to the ABCDE approach relies on the appropriate knowledge as a prerequisite. When knowledge is insufficiently acquired or retained, it can partially account for the incomplete or incorrect application [11-14]. Therefore, this research aimed to evaluate healthcare professionals' knowledge level of the ABCDE approach in Saudi Arabia.

There are large efforts to increase the utilization of the ABCDE approach, but adherence awareness and knowledge were suboptimal [8,9]. Similarly, our study revealed that the total knowledge score of the healthcare professionals was, on average, 52.94%. These findings indicate that the healthcare professionals in Saudi Arabia, especially in Taif, may need further awareness programs and training on the ABCDE approach.

In contrast, another study conducted among healthcare providers in the Netherlands found that the overall knowledge average score among healthcare providers was 80.1%. The variations between the results may be attributed to the difference between working experience [10].

Limited studies assessed the knowledge level of healthcare professionals regarding the ABCDE approach. Previous studies evaluated primary survey knowledge in life support courses, but not specifically ABCDE strategy [15-17]. This is the first study to evaluate the theoretical knowledge of the ABCDE approach among various healthcare professionals in Saudi Arabia at a random time.

In the present study, the knowledge score was significantly higher among males (56.37%) compared to females (49.85%). On the other hand, the average knowledge score was significantly higher among medical specialists (63.308%) and residents (55.67%) compared to nurses (46.34%). Similarly, another study found that the residents and medical specialists had significantly higher total scores than nurses [10]. A study by Linders et al. revealed that nurses showed lower approach adherence during a neonatal advanced life support course than residents and specialists, similar to our present study [9].

Other factors affecting our participants' knowledge level were age and working years. It was revealed that with increasing age, the total knowledge was significantly increased. Senior participants with more clinical experience may justify their actions and score higher due to frequent ABCDE approach use, which may justify their actions. In contrast, a study by Schoeber et al. found that younger participants had higher scores than older participants [10]. Furthermore, in the present study, increasing years of experience yielded higher total knowledge among participants. Similarly, Linders et al. demonstrated that the amount of experience of the healthcare professional significantly affected the total score [18]. Moreover, the difference in the results between studies may be explained by the participants' exposure level to critically ill patients.

Regression analyses revealed that the advanced trauma life support course and theoretical lectures significantly impacted the total knowledge score among the participants. Our results emphasize the importance of introducing such courses to healthcare professionals, especially in the ED, to raise their knowledge.

Limitations in this study included, firstly, its location in a specific region of Saudi Arabia, which may limit how applicable the findings are to other healthcare settings. Secondly, the study focused on assessing theoretical knowledge of the ABCDE approach, and the actual implementation of the approach in clinical practice was not evaluated. Therefore, the correlation between knowledge level and clinical performance remains uncertain.

## Conclusions

The ABCDE approach was valuable for initial patient assessment and treatment in acute medical and surgical emergencies. The findings indicate that there is a need for further awareness programs and training on the ABCDE approach, as the total knowledge score among healthcare professionals was found to be suboptimal. Several factors were associated with higher scores, including age, gender, working experience, profession category, and attending advanced trauma life support courses and theoretical lectures. Further research is needed to assess the association between knowledge level and clinical performance in different healthcare settings within Saudi Arabia. In addition, it could focus on evaluating the impact of interventions and educational initiatives on improving knowledge, adherence to the ABCDE approach, and patient outcomes.
